# Recent advances of automated methods for searching and extracting genomic variant information from biomedical literature

**DOI:** 10.1093/bib/bbaa142

**Published:** 2020-08-07

**Authors:** Kyubum Lee, Chih-Hsuan Wei, Zhiyong Lu

**Affiliations:** National Center for Biotechnology Information

**Keywords:** literature mining, genomic variant, mutation, literature search, named-entity recognition, named-entity normalization

## Abstract

**Motivation:**

To obtain key information for personalized medicine and cancer research, clinicians and researchers in the biomedical field are in great need of searching genomic variant information from the biomedical literature now than ever before. Due to the various written forms of genomic variants, however, it is difficult to locate the right information from the literature when using a general literature search system. To address the difficulty of locating genomic variant information from the literature, researchers have suggested various solutions based on automated literature-mining techniques. There is, however, no study for summarizing and comparing existing tools for genomic variant literature mining in terms of how to search easily for information in the literature on genomic variants.

**Results:**

In this article, we systematically compared currently available genomic variant recognition and normalization tools as well as the literature search engines that adopted these literature-mining techniques. First, we explain the problems that are caused by the use of non-standard formats of genomic variants in the PubMed literature by considering examples from the literature and show the prevalence of the problem. Second, we review literature-mining tools that address the problem by recognizing and normalizing the various forms of genomic variants in the literature and systematically compare them. Third, we present and compare existing literature search engines that are designed for a genomic variant search by using the literature-mining techniques. We expect this work to be helpful for researchers who seek information about genomic variants from the literature, developers who integrate genomic variant information from the literature and beyond.

## Introduction

Due to advances in genomic technology, such as next-generation sequencing and high-throughput methods, a large amount of data with genomic variant information is generated each day. As we are entering the era of precision medicine, this genomic variant information is becoming increasingly important as a means to develop personalized treatments for patients based on their genomic profile [1]. Genetic testing is already being used for clinical decisions, and, thus, obtaining information for the interpretation of the genomic variants of each patient is essential, as the differences among the genetic profiles of patients may cause different reactions to treatments, even when they share similar symptoms [2]. In addition, genomic mutations are key to understanding certain phenotypes, including oncogenic/signaling pathways, or to finding the target for immunotherapy [3, 4]. The biomedical literature is a rich resource for obtaining key information about genomic variants because most of the new findings in biomedical research are released and shared through peer-reviewed journals or conference publications and indexed in literature databases, such as PubMed [5] or PubMed Central (PMC).

**Figure 1:**
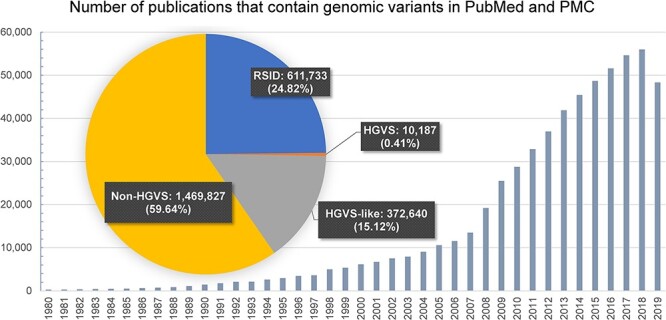
Bodies of literature that contain genomic variants in PubMed and PMC Open Access Subset (Accessed in February 2020). Normalized forms of genomic variants (RSID + HGVS) are only 25%. Please note that due to the PMC embargo policy, some of the full-text articles of 2019 are unavailable.

Biomedical information from various literature databases is organized and structured into knowledgebases to allow researchers to more easily access and search knowledge without having to read a vast amount of literature. Most knowledgebases are manually constructed by domain expert curators. After curators find and read publications, they extract information and store it in a knowledgebase. There are several excellent manually constructed genomic variant knowledgebases, including COSMIC [6] and the Genome-Wide Association Study (GWAS) Catalog [7]; however, as previous research has shown, this manual curation process in the biomedical field is often not scalable [8–10] because the manual curation process requires many highly trained domain experts and is expensive and time consuming. Because there are a large number of articles published every day, it is nearly impossible to read them all. Instead, the curators usually use a PubMed query with specific biomedical concepts (e.g. gene, disease, variant names) or keywords to find the candidate papers that are likely to include the information of interest. Considering that more than 3000 biomedical publications are indexed in PubMed every day, on average, and the number of publications that contain genomic variants is increasing every year, as shown in Figure 1, it is increasingly difficult to have enough expert curators to read all of the publications and to find all of the relevant information to include in the knowledgebases. Due to these limitations, many of the known and published genomic variant information are missing in many of the knowledgebases [11–13]. For example, Wagner *et al.*[13] combined six well-known cancer genomic variant knowledgebases, including Cancer Genome Interpreter (CGI), Clinical Interpretations of Variants in Cancers (CIViC), Jackson Labs Clinical Knowledgebase (Jax-CKB), MolecularMatch, OncoKB and the Precision Medicine Knowledgebase (PMKB), but found that only eight variants exist in all of the knowledgebases, which shows that many of the knowledgebases are missing the known genomic variant information. This limitation of knowledgebases shows that a literature search is still a very important source to obtain information on genomic variants.

The literature search for genomic variants can be difficult due to the various notations of genomic variants used in the research community; specifically, many of the genomic variants are written in multiple forms. The most popular form of substitution variant nomenclature by researchers consists of the location of the mutation, the wild type and mutated type of nucleotides or amino acids and one letter in front of the notation to distinguish whether it is a protein or a DNA-level change. For example, if valine (V as a symbol) is changed to glutamine (E as a symbol) at position 600 of an amino acid sequence in the *BRAF* gene, it is referred as ‘*BRAF* p.Val600Glu’ or ‘*BRAF* p.V600E’.

Recent Human Genome Variation Society (HGVS) nomenclature recommends the use of a reference sequence (RefSeq) ID and a version with variant nomenclature to avoid ambiguity [14]. Because the location number in the variant nomenclature can be different, depending on the reference sequence, it is necessary to specify this RefSeq information with variant nomenclature. Instead of using reference sequence ID from NCBI Reference Sequence Database (RefSeq ID) with variant notation, using reference single nucleotide polymorphism (SNP) ID (RefSNP ID or RSID), which is a locus accession ID, is another way to notate the location of genomic variants without ambiguity. RSID is fewer than nine digits of an integer number, and ‘rs’ in front of the numbers shows that it is an RSID (e.g. rs113488022). RSID is a simple way of pointing out genomic variants without ambiguity even though the RefSeq ID or gene name is not given. dbSNP/National Center for Biotechnology Information (NCBI) assigns RSID for variants. They also update, merge, and sometimes delete some RSIDs [15]. The manually curated information for each RSID is available in the dbSNP database (https://ncbi.nlm.nih.gov/snp). Due to the convenience of using RSIDs, many of the genomic variant databases, such as ClinVar and RefSeq, or many genome-wide association study databases (e.g. the GWAS Catalog) use RSIDs for genomic variants. Since 2018, however, RSID is used only for human variants but not for other species.

Following these standard nomenclatures for normalized forms of genomic variants is important, especially for a literature search and digital data-sharing purposes. The various forms of genomic variants make it more difficult to locate the information in the literature using information retrieval or other automated computational methods. Currently, much of the data are shared, transferred, combined, analyzed and reproduced in a (semi-)automated way for searching the information from the literature or performing high-throughput bioinformatics analysis. Even though non-normalized forms of variants are usually understood by human experts, they may not be understood by machines due to the ambiguity or variety of forms, which can cause errors or misinterpretations of given information [16]. The importance of using standard variant nomenclature has been raised in several studies [13, 17, 18]. Berwouts *et al.* [19] reported, however, that only 6% (13/216) of labs reported the genomic variations in HGVS nomenclature in 2010 [19]. Other publications also show that lab reports do not follow HGVS nomenclature and do not specify the reference sequence and version numbers correctly [20, 21]. Most of these results, however, are limited to cases of lab reports.

The pie chart in Figure 1 shows the percentage of the usage of standard genomic variant notations in publications in our analysis of the problem in the entire PubMed and PMC Open Access Subset [22]. Since dbSNP introduced reference SNP ID (RSID) in 1999 [15] and the variant nomenclature was proposed by HGVS in 2000, the use of HGVS format and RSID has increased substantially in recent years. Nevertheless, we see that more than 76% of the genomic variants in the publications are still in non-normalized forms. These publications have several forms of variants, such as ‘Glycine to Valine in 12’, ‘12G-->V’ and so forth. Further, these non-standard variations are not only ambiguous but also have a high probability that they are not searchable in the literature search engine.

**Table 1:** Genomic variant notations found in the bodies of literature in PubMed and PMC Open Access Subset^*^ (Standard nomenclatures are highlighted with gray)

Type	Example	Numbers	Percentage
WNM	V600E	1 107 672	44.95
RSID	rs113488022	611 733	24.82
c.Na>a	c.1799T>A	135 510	5.50
p.WNM	p.V600E	105 921	4.30
Na>a	1799T>A	98 228	3.99
p.WwwNMmm	p.Val600Glu	79 038	3.21
WwwNMmm	Val600Glu	57 386	2.33
_to_	Val to Glu	54 125	2.20
c.Ntype1	c.76delA	43 648	1.77
Ntype1	76delA	21 992	0.89
Deletion (without genotype)	76del	17 481	0.71
_with_	V600 with Glu	9581	0.39
_for_	Thr for Ile-142	8342	0.34
_by_	Val 600 by Glu	7762	0.31
_at_	mutation at V600	7728	0.31
c.WNM	c.T1799A	6150	0.25
ref:c.Na>a	NC_000023.10:g.33038255C>A	4676	0.19
WNfs	A456fs	3819	0.15
Insertion (without_genotype)	76_77ins	2818	0.11
No-Location_W/M	T/A	2487	0.10
p.WwwNtype	p.Arg456fs	2373	0.10
ref:p.WNM	NP_003997.1:p.T24C	2350	0.10
Www-N-Mmm	Val-600-Glu	2265	0.09
_of_	serine96 of histidine	2094	0.08
_in_	mutation in 600	1977	0.08
stop	Trp149Stop	1967	0.08
ref:p.WwwNMmm	NP_003997.1:p.Val600Glu	1856	0.08
W>M (without location)	T>A	1305	0.05
ref:c.Ntype2	NM_018136.4: c.10013delA	1305	0.05
Na>a	504g>a	1164	0.05
WwwNfs	Arg456fs	602	0.02

*Accessed in February 2020. *Not all the examples are the same variant.

**Figure 2:**
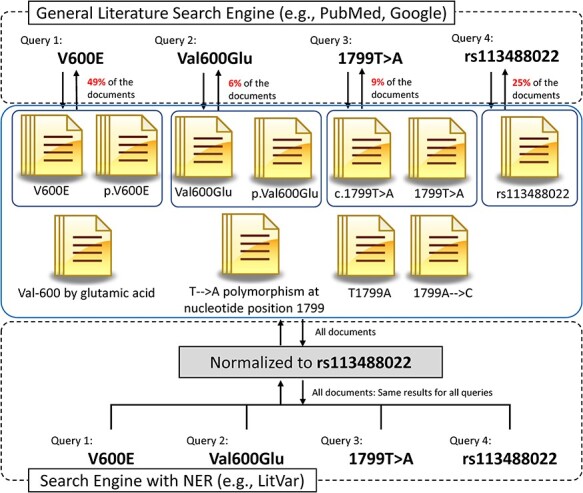
Comparison between general search engine and search engines with NER for genomic variant search. Due to various notations of genomic variants, general search engines typically need to use multiple queries to find relevant publications. The NER module, however, can find and normalize genomic variants from publications and query inputs and thus can provide more complete and precise search results.

Table 1 shows how many different forms of genomic variants are found in scholarly articles. We analyzed the publications in PubMed and found a total of 53 different variant notations to describe the ‘p.G12V’ mutation, including two HGVS standard formats. Due to these non-normalized genomic variant notations in the literature, there is a high probability that many of the publications are not included in the results of a single genomic variant keyword search. For example, most PubMed users use the simple ‘wild type amino acid (w) + location number (N) + mutated type amino acid (m)’ (wNm) form of genomic variants for a query (e.g. V600E). In this case, the query results show less than 49% of all the publications that have relevant information. Further, 51% of the publications that use different notations (e.g. rs113488022, Val600Glu, Val 600 by Glu) are not shown in the query results (Figure 2). Unless users query using multiple genomic variant forms, these publications have the potential to be non-searchable in literature search engines.

To overcome the lack of information in manually curated knowledgebases and the query mismatching problem in the general literature search engines, the use of automated genomic variant finding, using text mining techniques, has been highly researched [23]. There are a number of literature-mining tools that find mutation mentions from the bodies of literature and normalize them into standardized forms. Next, we look into various literature-mining techniques that address this problem through the recognition and normalization of various forms of genomic variants in the literature. Subsequently, we review the publicly available literature search Web services that are specifically designed for genomic variant search by adopting the literature-mining techniques.

## Text-mining techniques and tools for variant recognition and normalization

### Named-entity recognition and named-entity normalization

Named-entity recognition (NER) is a process to identify the biomedical entities mentioned in the text. In biomedical literature, finding genes, diseases, drugs or genomic variant names is key to achieving better performance in literature mining or information retrieval. Whereas NER is usually described as identifying the mentions of the entities, named-entity normalization (NEN) requires mapping the recognized entity to a certain ID or concept so that different forms of the same entities can be recognized as the same concept. For example, after ‘Breast tumor’, ‘Mammary cancer’ and ‘Neoplasm of breast’ are recognized as diseases in the literature by the NER process, they should be mapped to the same concept (MESH ID: ‘D001943’) by the NEN process. As described in the previous section, a biomedical entity, especially a genomic variant, may have multiple name forms in the literature, thus NER and NEN are important to identify them and to map them to the corresponding concept.

Many of the NER methods for biomedical entities, such as genes, diseases and drugs, partially (or fully) rely on prebuilt dictionaries. Most of the genomic mutation NER processes, however, are done using a text-mining technique called regular expression. Regular expression is a method to define a string pattern to find certain forms of character sequences. It is easier to manually construct rules to distinguish genomic variants using regular expression not only because there are so many genomic variants, and it is difficult to build a dictionary, but also because most genomic variants are described in certain formats that are expressed in several patterns. Figure 2 shows that there are many different notations for the same genomic variants and how NER/NEN helps the genomic variant extraction. During this NER process, most of the recognition tools distinguish the type of mutation (e.g. substitution, deletion, insertion), wild type and the mutated sequence of the variant. Without gene name or the sequence ID, however, this information is still ambiguous for users to be able to specify certain variants. Many of the NER of genomic variants use gene name NER to find the gene with which the genomic variant is associated.

After the NER process is used to recognize the genomic variants, some of the existing tools can normalize (NEN) the variants into RSIDs. For example, tmVar 2.0 [24] and SETH [25] recognize not only variants but also gene names that appear near the variants in the text. They link the gene names to variants so that the tool can find gene-variant pairs to specify the correct variant information. These tools use the gene-variant information to query external databases, such as dbSNP, to find the HGVS form or RSID of the genomic variant. After using the NEN process, it is easier to locate certain variants from the literature without being concerned about the various forms of variants.

### Genomic variant NER and normalization tools

There are several genomic variant NER/NEN tools that are publicly available. Most of the tools are based on a set of regular expressions to recognize the genomic variants, while others are built based on machine learning.

MutationFinder [26] is a regular expression-based mutation NER tool. Based on the six rules that the authors found to yield good precision and recall of recognizing mutations from the text, MutationFinder uses ~700 regular expressions to recognize not only point mutations with wild-type residue + amino acid position + mutant residue (wNm – e.g. V600E) forms but also the mutations in simple natural language forms (e.g. valine 600 substituted with glucose). MutationFinder first splits the input text into a sentence level and then applies the regular expressions to extract mutations from each sentence. The source code is available in Java, Perl and Python. MutationFinder focuses only on recognizing mutation names and does not find associated genes for mutation. The source codes, however, can be combined into another system and used as a part of other bigger systems to recognize a mutation–gene or mutation–gene–disease relation [25, 27, 28].

Extractor of Mutation (EMU) [29] is another regular expression-based mutation extraction tool that can find not only point mutations but also insertion or deletion mutations. After finding mutations, EMU uses another set of regular expressions to filter out false-positive mutation terms. EMU also finds the gene name with which the mutation is associated. When EMU recognizes a mutation, it also finds all of the gene names that occur in the same text as candidates, using the human gene name list from HUGO and the NCBI databases. After finding the candidate genes for mutations, EMU retrieves each of the candidate genes’ sequences from the NCBI RefSeq (https://www.ncbi.nlm.nih.gov/refseq/) database. Using the retrieved sequence, EMU finds the matching genes that have the same information about the mutation’s wild type and the location information. For example, to validate the candidate pair of *BRAF*-V600E, EMU finds the amino acid sequence of *BRAF* from the NCBI RefSeq database and validates whether the 600th amino acid is V. EMU codes are available in Perl and consist of two parts: NER and gene sequence verification. Using the same EMU tool, the authors extended their work by combining EMU results with crowdsourcing [30]. For this extended project, they used GenNorm [31] instead of their own gene NER tool.

SETH [25] supports both NER and NEN for genomic variants. First, for NER of genomic variants, SETH uses extended Backus–Naur form and the regular expressions from MutationFinder [26] together to find genomic variants. Also added was a set of regular expressions to recognize deletions, insertions, additional substitutions, frameshift mutations and RSIDs. SETH uses some heuristics to find the gene that is related to the genomic variants using GNAT [32], which is a gene name recognition tool. As does EMU, SETH uses the sequence of the genes to verify that the mutation resides in the gene. The tool uses UniProt and dbSNP as the external source to retrieve the sequence of the genes to validate the gene-variant mapping. It returns the normalized genomic variants as the HGVS form, UniProt ID or RSID. Mapping the genomic variants to RSID removes the ambiguity of the variants so that users can easily find the right mutation information from the external databases. The Java codes for SETH are publicly available.

tmVar 2.0 [24] is an updated version of tmVar [33] and uses conditional random fields (CRF) with regular expression to find genomic variants and to map the variant with a related gene that is recognized using the GnormPlus [34] NER tool. tmVar 2.0 also supports NEN, which normalizes the recognized gene and genomic variant pair into RSID. The Java code of tmVar 2.0 is publicly available. tmVar 2.0 is also a part of PubTator Central [35], which is a web-based system that provides automatic annotation of biomedical concepts in PubMed and PMC articles; the tmVar 2.0 results for these articles are available at the tool’s website or through its RESTful API.

AVADA [36] employs machine learning and regular expressions for improved gene-variant mapping in full-text articles. It uses 47 regular expressions to recognize genomic variants. It also employs a custom-built gene name recognition tool that learns and uses lists of gene names obtained from the HUGO Gene Nomenclature Committee (HGNC) and the UniProt database for finding candidate gene names associated with variants in an article. As do EMU and SETH, AVADA retrieves sequences of the candidate genes from the RefSeq database and uses the results for finding the gene-variant mapping. Finally, it uses a machine-learning classifier (GradientBoostingClassifier) to find the related gene for each genomic variant. The Python codes of AVADA are available with a list of automatically retrieved variants from the full-text literature.

Table 2 provides a summary of the main functions and the availability of the tools that are introduced above.

**Table 2:** Comparison of genomic variant NER tools (wNm: w as the wild residue, m as the mutated residue and N as the location of the mutation)

Tool	Description	Method	Source code	Target	Normalization	Year released	URL
MutationFinder [26]	Recognizes multiple forms of mutations, including natural language forms	Regular expression	Python, Perl, Java	Point mutations	wNm	2007	http://mutationfinder.sourceforge.net/
EMU [29]	Contains validation filter, uses outside database and uses manually built exception list	Regular expression, protein sequence filter	Perl	Point mutations, insertions, deletions, mutation–gene	Gene name + wNm	2011	http://bioinf.umbc.edu/EMU/ftp
SETH [25]	Finds mutation, maps it to gene and normalizes it to RSID	Extended Backus–Naur Form (EBNF), grammar and MutationFinder	Java	Mutation–gene pairs, RSIDs	RSID, or gene name + HGVS-like	2016	https://rockt.github.io/SETH/
tmVar [24, 53]	Covers most of the mutation forms; tmVar 2.0 is updated from tmVar, maps the gene, and normalizes it to RSID	CRF, regular expression, GNormPlus	Java, RESTful API	Mutation–gene pairs, RSIDs	RSID, or gene name + HGVS-like	2013, 2017	https://www.ncbi.nlm.nih.gov/research/bionlp/Tools/tmvar/
AVADA [36]	Contains validation filter, uses outside database and maps it to RefSeq	Regular expression, GradientBoostingClassifier for mapping gene	Python	Mutation–gene pairs, RefSeq	HGVS (with-RefSeq)	2019	http://bejerano.stanford.edu/AVADA/
MuteXt [42]	Uses Swiss-Prot for validating the sequence and finding protein name for the mutation; uses manually built exception list	Regular expression, protein sequence filtering	N/A	Point mutations (wNm) with protein name	Gene name + wNm	2004	N/A
MEMA [41]	Recognizes 30 different patterns of mutation	Regular expression	N/A	30 different patterns of protein mutation/DNA mutation with gene name	Gene name + wNm	2004	N/A
VTag [47]	Uses CRF trained with 27 types of mutation with 63 421 features (a part of Biosfier)	CRF, regular expression	N/A	Point mutations, translocation, deletion	wNm	2004	N/A
Mutation GraB [43]	Upgraded version of MuteXt.(RegEx + filtering); uses graph-based bigram traversal to improve protein–mutation association	Regular expression, graph-based bigram traversal for protein mapping, protein sequence filtering	N/A	Protein point mutations (wNm) with protein name	Gene name + wNm	2007	N/A
MuGeX [48]	Able to find 20 patterns and generated regular expressions (Results are available)	Regular expression, Rocchio/naive Bayesian algorithm for disambiguation resolution (Nucleotide OR AA mutations)	N/A	20 Different patterns of protein–mutation with gene name	Gene name + wNm	2007	N/A
Yip *et al*. [45]	Suggests four patterns of protein point mutations, Swiss-Prot sequence filter	Regular expression, protein sequence filtering	N/A	Protein point mutations with protein name	Gene name + wNm	2007	N/A
mSTRAP [72]	Uses regular expression with ontology mining and reinstantiation, using protein structure	Regular expression, ontology-based methods, protein sequence filtering	N/A	Protein point mutations with protein name	Gene name + wNm	2007	N/A

To understand the pros and cons of different methods (regular expression versus machine learning), researchers have benchmarked and compared the tools with each other on multiple datasets that are publicly available [24, 26, 29, 37, 40, 53, 74, 75]. In Supplementary Table S1 available online at https://academic.oup.com/bib, we list publicly available genomic variant datasets that can be used for benchmarking the tools. Except for BRONCO [37] and SNP-Corpus [38], most of the datasets are published with the NER tools to test its performance. Lee *et al*. [37] and Yepes *et al*. [39] compare the genomic variant NER performance of the tools in multiple datasets. Supplementary Table S2 available online at https://academic.oup.com/bib shows the combined results from both articles in multiple benchmark datasets. As shown in the table, tmVar shows the highest F1 scores in three of the five datasets. It also shows the best precision on BRONCO and best recall on Variome [40] datasets, which are full-text corpora. The performance of tmVar 2.0 on the MutationFinder corpus is reported in [24] and shows better results as compared to the previous version. In the same publication, the NEN of tmVar 2.0 showed better performance compared to that of SETH. As shown in the above publications, the accuracy of the variant extraction tools is not yet perfect but is still precise enough to effectively find non-normalized variants in the literature.

In addition to these publicly available tools, there are a few others that are worth discussing. We list those currently unavailable variant NER tools at the bottom of Table 2 and briefly discuss them in this section.

MEMA [41] expanded the multiple regular expressions, as used by MutationFinder, to recognize 30 other patterns of mutations that are not the “wild-type amino acid–location–mutated type of amino acid” form. For example, MEMA can recognize mutations such as ‘Arg506 to Gln’, ‘valine 804$ \rightarrow $leucine’ and ‘677C$ \rightarrow $T, Methionine for valine at position 30’. MEMA finds the gene name for each mutation, using the HUGO database as a dictionary, and finds gene names from the abstract or a sentence in which that mutation is mentioned. If multiple candidate genes for a mutation are found, the gene that is mentioned most closely in the text is chosen.

Similar to EMU, there are several other tools that use sequence filtering to find gene names in which the target mutation resides. MuteXt [42] and MutationGraB [43] are early-stage, single-point mutation-extraction tools that use regular expressions. After finding mutation names using regular expressions, these tools also find candidate gene names in the text around the mutation and use an amino acid sequence to filter the gene names, as does EMU. Unlike EMU, both tools use Swiss-Prot (currently, UniProtKB [44]) data as a dictionary to find candidate protein names and find the protein sequence of the candidate genes for validating the protein–mutation pairing. MutationGraB uses a graph bigram method to improve the mutation–protein mapping performance. Yip *et al*. [45] summarized four different patterns of mutation notations found in the literature and built regular expressions to recognize them. They also added a vocabulary set (e.g. polymorphism, amino acids) to find sentences that describe mutation events.

DiMeX [46] uses multiple regular expression patterns to detect mutations, including protein/DNA-level mutations, insertions, deletions and SNP IDs, as well as natural language forms of mutations as do MutationFinder and tmVar. DiMeX also finds associated genes and diseases with the mutation that are recognized by analyzing the sentence structures. DiMeX results are available at its website, but the method is not available.

Just as tmVar uses machine learning for its NER process, other tools also use machine learning for their NER process. VTag [47] used CRFs to recognize mutation patterns with the event type (e.g. point mutation, translocation, deletion). MuGeX [48] used machine learning to solve disambiguation problems in mutation NER. For example, it is difficult to determine whether some mutations such as ‘A14C’ are an amino acid mutation (p.Arg14Cys) or a DNA-level mutation (c.14A>C). In addition, ‘T47D’ is a cell-line name but looks like a mutation notation. MuGeX uses a naïve Bayes algorithm and a Rocchio algorithm to determine the correct mutation types of these terms.

## Literature search engines designed for genomic variant search

As noted, general literature search engines, such as PubMed, are not suitable for a genomic variant search because there are many forms of genomic variants in the literature. Regular literature search engines usually use keyword matching for a literature search, and the search engine returns results only when the words in a literature exactly match the query words (or parts of the query words). Sometimes, query expansion (QE), an information retrieval technique, is used to change the query (e.g. adding synonyms to the query) for improving search quality. Currently, PubMed uses QE for biomedical entities in MeSH (e.g. diseases, chemical names and so on) [49]; however, it does not have QE support for genomic variants.

To overcome the problem of mutation query in a literature search engine, some of the biomedical literature search engines adopt the NER and NEN methods for their system to normalize the genomic variants in the literature and the user input query.

LitVar [50] is a literature search tool for variant publications that was developed based on a literature-mining technique. LitVar uses tmVar 2.0 [24] for NER and NEN of the genomic variants for the literature and the user input query. LitVar regularly indexes the articles in PubMed and the PMC Open Access Subset, finds genomic variants from the articles and normalizes them into RSIDs. When a user types a genomic variant as a query, tmVar 2.0 finds the genomic variant from the query and normalizes it into RSID. In the event that a user query is ambiguous due to a missing gene or RefSeq information, LitVar also suggests a gene that is paired mainly with the mutation in the literature so that users can see only the specific mutation result in which they are interested. As the genomic variants in both the input query and the indexed literature are normalized into RSIDs, LitVar can match the query and the articles without the keyword mismatching problems that occur in regular literature search engines. Further, more comprehensive results of the genomic variant search are provided. For example, when users query ‘G12V’ without a gene name, which is considered an ambiguous query for genomic variants due to the missing gene name or RefSeq information, LitVar finds the most popularly used G12V in the literature, which is *KRAS* p.G12V, and normalizes the query into ‘rs121913529’, which is the matching RSID for the genomic variant. (If the user wanted to see the G12V of other genes, such as *HRAS* p.G12V (rs104894230), LitVar also shows an option that allows users to switch to the *HRAS* results.) During the indexing time of the LitVar server, LitVar finds all possible forms of *KRAS* p.G12V that appear in the literature, such as ‘Gly12Val’, ‘Gly12 --> Val’ and even ‘glycine to valine substitution at position 12’, normalizes them into RSIDs and saves them in the server. These preprocessing and indexing processes, using NER and NEN, make it possible for LitVar to provide comprehensive results for a genomic variant literature search. LitVar also provides RESTful API so that users or developers can easily utilize LitVar results for their own purpose. LitVar is updated monthly for newly published articles in PubMed and the PMC Open Access Subset.

Similar to LitVar, Variant2Literature [51] is a literature search system designed for a genomic variant search. It also uses tmVar and GnormPlus for gene and mutation NER and targets all of the literature in PubMed and PMC, as does LitVar. Variant2Literature finds variants from not only the abstract and the full-text part of the literature but also supplementary files that are in XML, PDF, DOCX, DOC, XLSX and CSV formats. The authors of the system also used an image processing technique to extract variants from the tables in the literature in PDF formats. They performed the comparison test and, by using the supplementary files and the image processing, increased the recall more than two times (43.47–98.38%) in their own test dataset. Variant2Literature supports Variant Call Format files for input and output of the system, which is widely used in bioinformatics tools.

VIST [52] is a variant search tool for precision oncology with a machine learning-based ranking method. It uses PubTator API [53] to obtain the NER information of genes, chemicals and genomic variants from the PubMed abstracts. This tool uses ranking functions to score each publication using three different scores, CancerScore, ClinicalScore and TypeScore, which are used to measure how much a publication is related to cancer, clinical use and specific cancer type, respectively. For the ranking function, support vector machine models, using multiple datasets, were trained. This machine learning-based ranking method enables clinical researchers to easily find the clinically relevant publications on the queried genomic variants. VIST also provides the search results of ClinicalTrials.gov [54], which is the database of clinical studies around the world.

BEST [55] and GeneView [56] are biomedical literature search engines designed for searching biomedical entities, including genes, drugs, diseases and genomic variants. They find genomic variants, using NER tools from PubMed abstracts, and highlight them when the users search for those variants. BEST uses tmVar [33] for the mutation NER process. GeneView used MutationFinder [26] for the earlier version of the service and uses SETH [25] for the current version. As shown in Table 3, however, the query results of ‘p.V600E’ and ‘V600E’ in both engines are different, which means that they do not support NER for input queries. Each service has its own ranking function. BEST uses the journal’s reputation and publication date for the ranking for the results, and GeneView uses section boosting (e.g. if the target term is in the title or abstract section, the document gets a higher score) with the publication date. BEST is updated daily, but GeneView has not been updated since 2017.

**Table 3:** Automated genomic variant literature search engines and a comparison of the results with PubMed and PMC

Search Engine	Variant normalization	QE (normalizes the query)	Content scope	Evaluation				URL	Publicly available?	Last update^a^
				‘*BRAF* p.V600E’ results^a^	‘*KRAS* p.G12D’ results^a^	‘*XRCC2* p.R188H’ results^a^	‘*KCNH2* p.L552S’ results^a^			
LitVar [50]	tmVar, GNormPlus	Yes	Title, abstract, full text (PubMed and PMC Open Access Subset)	13 987	4940	110	12	https://www.ncbi.nlm.nih.gov/CBBresearch/Lu/Demo/LitVar	Yes	May 2020
Variant2Literature [73]	tmVar, GNormPlus	Yes	Title, abstract, full text, supplementary (PubMed and PMC Open Access Subset)	8473	3318	108	9	https://variant2literature.taigenomics.com	Yes	February 2020
VIST [52]	tmVar, GNormPlus (Through PubTator)	No	Title, abstract (PubMed)	111 (*BRAF* p.V600E) 2898 (*BRAF* V600E)	*33 (*KRAS* p.G12D)* *519 (*KRAS* G12D)*	0 (*XRCC2* p.R188H) 21 (*XRCC2* R188H)	0 (*KCNH2* p.L552S) 5 (*KCNH2* L552S)	https://vist.informatik.hu-berlin.de	Yes	2018
BEST [55]	tmVar	No	Title, abstract (PubMed)	133 (*BRAF* p.V600E) 3224 (*BRAF* V600E)	*35 (*KRAS* p.G12D)* *556 (*KRAS* G12D)*	0 (*XRCC2* p.R188H) 21 (*XRCC2* R188H)	0 (*KCNH2* p.L552S) 3 (*KCNH2* L552S)	http://best.korea.ac.kr	Yes	May 2020
GeneView [56]	SETH and GNAT	No	Title, abstract, full-text (PubMed and PMC Open Access Subset)	45 (MUTATION:p.V600E AND ENTREZ:673) 1010 (MUTATION:V600E AND ENTREZ:673)	4 (MUTATION: p.G12D AND ENTREZ: 3845) 40 (MUTATION: G12D AND ENTREZ: 3845)	1 (MUTATION:p.R188H AND ENTREZ:7516) 51 (MUTATION:R188H AND ENTREZ:7516)	2 (MUTATION:p.L552S AND ENTREZ:3757) 5 (MUTATION:L552S AND ENTREZ:3757)	http://bc3.informatik.hu-berlin.de/	Yes	2017
Mastermind [57]	Unknown	Yes	Title, abstract, full text (Unknown)	20 047	5568	229	47	https://mastermind.genomenon.com/	Limited	May 2020
*PubMed*	*N/A*	*No*	*Title, abstract*	*146 (*BRAF* p.V600E)* *4402 (*BRAF* V600E)*	*49 (*KRAS* p.G12D)* *1004 (*KRAS* G12D)*	0 (*XRCC2* p.R188H) 21 (*XRCC2* R188H)	0 (*KCNH2* p.L552S) 5 (*KCNH2* L552S)	https://www.ncbi.nlm.nih.gov/pubmed	*Yes*	*Updated daily*
*PMC*	*N/A*	*No*	*Title, abstract, full text*	*446 (*BRAF* p.V600E)* *11918 (*BRAF* V600E)*	*323 (*KRAS* p.G12D)* *4603 (*KRAS* G12D)*	1 (*XRCC2* p.R188H) 54 (*XRCC2* R188H)	2 (*KCNH2* p.L552S) 6 (*KCNH2* L552S)	https://www.ncbi.nlm.nih.gov/pmc	*Yes*	*Updated daily*

^a^Evaluated in May 2020.

Mastermind [57] is a commercial genomic variant search engine and uses NER and NEN for both the query and literature to provide more comprehensive results. Mastermind provides several scoring functions, such as publication date, relevance, journal name or the impact factor of the journal, to rank the publications in the result. However, many of the services are not fully available to non-paid users, and only some of the literature (100 results per query) in the search results are viewable. Also, Mastermind provides ACMG interpretation, clinical significance and genetic mechanism analysis results for the input genomic variant, but these functions are also available only to paid users.

Table 3 shows the comparison of the literature search engines that we introduced above for a few example variants of various popularity in the literature. The table also shows the mutation query results of each search engine. Note that the query results might contain false positives. Hence, the performance of the search engines in Supplementary Table S2 available online at https://academic.oup.com/bib should be considered when interpreting the number of returned results in Table 3. In general, the search engines that support QE (variant normalization in the query), such as LitVar, Variant2Literature and Mastermind, find many more publications as compared to other search engines. For example, in PubMed and PMC, the ‘*BRAF* V600E’ query returns 4402 and 11 918 results, respectively. However, LitVar returns 13 987 results, which is the highest number out of all of the public search engines. In addition, some of the other search engines show different results for ‘V600E’ and ‘p.V600E’ queries, when the NER-based search engines show the same results. This example shows why users need to use a specific literature search engine, instead of PubMed or PMC, for genomic variants.

## Discussion and conclusions

Considering that the text-mining approaches are not yet perfect, it is highly recommended that authors use the correct notation of the genomic variants so that they can be found even without the help of these text-mining tools [14, 18, 19, 23, 58, 59].

Even though authors would like to use the correct form of genomic variation for their manuscript, sometimes it is difficult to find the correct notation, and it can be easy to make mistakes, such as using wrong RefSeq ID, location number or incorrect grammar for the HGVS format [60]. To make it easier for users to use the correct notation of genomic variants, there are some online verification resources available. As den Dunnen [61] suggested, uploading the variants information to databases such as ClinVar [62] or the Leiden Open Variation Database [63] before publication submission would be helpful as a means to find the correct form of normalized genomic variant notations. In this way, during the variant submission process, the authors are reassured that they are using the correct HGVS format of genomic variants with the right RefSeq information.

In addition, converting and submitting unstructured genomic variant information (such as the literature) into a structured form in ClinVar will make it easier for other researchers to find the information and to find it even earlier than the publication of the information. If submitting the genomic variants to these databases is not possible, there are some tools that help to verify the standard notation of the genomic variants. For example, VariantValidator [64], which is a web-based service of the HGVS Python package [65], suggests the correct format for variants and validates the HGVS format of the mutation. Mutalyzer [66] is another Web-based tool for HGVS nomenclature verification and provides an HGVS syntax checker, SNP converter and reference sequence information for genomic variants.

In addition, as many publishers, conference organizations, research institutions and funding agencies have policies for publication, they may require researchers to upload their information on genomic variants to databases before they submit their work to journals or conferences and to use HGVS nomenclature for all variant-related papers.

Despite these author-assisting tools, non-standard notations are still widely used in the literature. These non-standard notations are sometimes not searchable in PubMed-like systems and are often ambiguous. These non-searchable and ambiguous genomic variant problems are critical during the information retrieval or high-throughput analysis, using the literature. For these reasons, using normalized genomic variants, such as HGVS nomenclature, or RSID for biomedical literature is highly recommended.

While normalization of variants with a unique genomic position greatly improves search, it is important to note that variants of the same locus may have different functional consequences or phenotypes depending on the variant allele expressed. As with SNP RS334, the nucleotide change A->T is prevalent for sickle cell anemia [67], while A->G is less prevalent with unknown clinical significance. Further enhancement of variant detection and normalization methods is needed for obtaining more precise search results in future research.

In this survey, we have reviewed a number of NER/NEN tools, such as tmVar or SETH, as well as genomic variant-specific literature search tools, such as LitVar. These developments allowed much-improved search results for extracting and finding genomic variants compared to that of PubMed. These search engines also provide disease and drug information that occurs in the same literature, as found by the query. With research advances in automated information extraction and retrieval methods, such as advanced deep learning techniques [68–71] for biomedical text mining, we expect to see continued developments and improvements for the unmet needs of variant information access in precision medicine and cancer research.

**Key Points**

- By analyzing the written patterns of genomic variants in PubMed articles, we show the problem of non-standard description of genomic variants.
- We survey how text-mining techniques can help and review a comprehensive list of genomic variant named-entity recognition and normalization tools.
- We survey and compare the functionality and availability of different literature search systems for variant search.

## Supplementary Material

SuppTables_bbaa142

## Notes

**Kyubum Lee**, PhD is a postdoctoral fellow at National Center for Biotechnology Information, working on biomedical literature mining using machine learning.

**Chih-Hsuan Wei**, PhD is a research scientist at National Center for Biotechnology Information, working on biomedical named-entity recognition.

**Zhiyong Lu**, PhD FACMI is a Senior Investigator and Deputy Director for Literature Search at NCBI/NLM where he leads text mining and machine learning research and directs overall R&D efforts to improve PubMed search and information access.
