# Supplementary material for: Recent advances of automated methods for searching and extracting genomic variant information from biomedical literature
Source: Brief Bioinform. 2020 Aug 7;22(3):bbaa142. doi: 10.1093/bib/bbaa142 (PMC8138883; doi:10.1093/bib/bbaa142)
Supplement: SuppTables_bbaa142 [file supptables_bbaa142.docx]

**Table S1. Genomic variant benchmark datasets**

| **Name** | **Target Text** | **Entity (Relations)** | **Normalization** | **Total Variants** | **Unique Variants** | **URL** |
| --- | --- | --- | --- | --- | --- | --- |
| Mutation Finder Corpus [26] | 508 Abstracts | Variant | -- | 482 | 451 | <http://mutationfinder.sourceforge.net/> |
| EMU Corpus [29] | 109 Abstracts | Gene-Variant  (2 Diseases: BC, PC) | Gene + mutation | 287 | 237 | http://bioinf.umbc.edu/EMU/ftp |
| tmVar corpus [53] | 500 Abstracts | Variant | -- | 1057 | 871 | <https://www.ncbi.nlm.nih.gov/research/bionlp/Tools/tmvar/> |
| tmVar 2.0 corpus [24] | 158 Abstracts | Variant | RSID | 432 | 285 | <https://www.ncbi.nlm.nih.gov/research/bionlp/Tools/tmvar/> |
| Variome [40] | 10 Full Text | Variant, Gene, Disease, patient | -- | 52 | 50 | N/A |
| BRONCO [37] | 108 Full Texts | Variant – Gene – Drug – Disease – Cell-line | Gene + HGVS(-like)  RSID (only 90/275) | 403 | 275 | <http://infos.korea.ac.kr/bronco/> |
| OSIRIS [74] | 105 Abstracts | Variants | RSID (only 109/264) | 374 | 264 | <https://sites.google.com/site/laurafurlongweb/databases-and-tools/corpora> |
| Nagel et al. [75] | Total 57 Abstracts (only 18 for mutations) | Variants | - | 70 | 48 | <http://bionlp-corpora.sourceforge.net/proteinresidue/> |
| SNP Corpus, Thomas et al. [38] | 295 Abstracts | Variants | RSID | 526 | 384 | <https://www.scai.fraunhofer.de/en/business-research-areas/bioinformatics/downloads/corpus-for-normalization-of-variation-mentions.html> |

**Table S2. Variant NER tools benchmark results (Combined results from Lee et al. [37] and Yepes et al. [39])**

| **Dataset** | **Mutation Finder** | | | **EMU** | | | **tmVar** | | | **BRONCO** | | | **Variome** | | |
| --- | --- | --- | --- | --- | --- | --- | --- | --- | --- | --- | --- | --- | --- | --- | --- |
| **Tool** | Precision | Recall | F1-score | Precision | Recall | F1-score | Precision | Recall | F1-score | Precision | Recall | F1-score | Precision | Recall | F1-score |
| MutationFinder [26] | **0.985** | 0.805 | 0.886 | **0.995** | 0.747 | 0.854 | **0.985** | 0.294 | 0.453 | 0.915 | 0.876 | **0.895** | 0.412 | 0.059 | 0.104 |
| EMU[29] | 0.977 | 0.801 | 0.880 | 0.956 | **0.959** | 0.957 | 0.845 | 0.699 | 0.765 | 0.773 | **0.903** | 0.833 | 0.725 | 0.559 | 0.632 |
| SETH [25] | 0.970 | 0.830 | 0.890 | 0.976 | 0.903 | 0.938 | 0.940 | 0.810 | 0.870 | 0.872 | 0.894 | 0.883 | **0.890** | 0.575 | **0.775** |
| tmVar [53]  tmVar 2.0 [24] | **0.985** | **0.842** | **0.908** | 0.988 | 0.952 | **0.970** | 0.955 | **0.937** | **0.946** | **0.938** | 0.844 | 0.884 | 0.757 | **0.686** | 0.720 |
